# To Float or Not to Float? Internal Migration of Skilled Laborers in China

**DOI:** 10.3390/ijerph17239075

**Published:** 2020-12-04

**Authors:** Yanqiao Zheng, Xiaoqi Zhang, Qiwen Dai, Xing Zhang

**Affiliations:** 1 Department of Finance, Zhejiang University of Finance and Economics, Hangzhou 310018, China; yanqiaoz@buffalo.edu; 2 National School of Development, Southeast University, Nanjing 210000, China; xiaoqizh@buffalo.edu; 3 School of Economics & Management, Guangxi Normal University, Guilin 541004, China; 4 Finance Department, Minzu University of China, Beijing 100081, China; 201805017@muc.edu.cn

**Keywords:** internal migration, skilled laborers, location choices, amenity, population redistribution

## Abstract

This paper uses data from job-recruiting platforms to study the distribution patterns and migration destination choices of a skilled internal migrant population. We find that, in most first-tier cities and most emerging second-tier cities, more than half of the skilled jobseekers do not hold local household registration. The most important finding of this paper is the heterogeneity of attributes prioritizations between intra- and inter- provincial migrants. Intra-provincial skilled migrants put more value on employment opportunities than on amenity attributes, while their inter-provincial counterparts prioritize amenity over employment aspects.

## 1. Introduction

As of the middle 2010s, there are about 250 million floating population in China, of which about 20% have college degrees [1,2,3,4]. The prominence of skilled floating population has attracted the attention of recent research [5,6].

The term “floating population” conjures up images of unsettled persons, and its definition can be ambiguous at times [7]. In fact, there is no official definition of this term in China, since China is the only country that routinely uses this term and the only country that has such an influential hukou system. Often, the “floating population,” or liudong renkou, refers to the large and increasing number of migrants without local household registration status, or hukou [8].

Since the late 1970s, China’s reform has made its population more mobile. People move from rural areas to urban regions in seek of higher wages and better living conditions. Meanwhile, since 1999, China has undergone a major higher education expansion at an unprecedented ten-fold scale [9]. As a result, the migration trend has continued with more educated young people moving from rural to urban parts, from small towns to megacities.

Floating population as a type of migrants are different from “permanent migrants” who have already transferred their place of registration to their current place of residence. Without local household registration status, the floating population have limited access to local health services, education, and social securities. Moreover, their offspring are also deprived of local registration status, even if born at the same place of residence. In this context, it is of importance to understand why, even not afforded full benefits of citizenship, the floating population keep floating.

At the society level, China’s large floating population brings different lifestyles and cultural values to their place of residence [10,11]. They are an indispensable force of economic development and cultural integration and will strongly affect both China’s patterns of urbanization and its population distribution among regions [8]. As a matter of fact, the floating population constitute over 25% of total population in most counties in the Yangtze River Delta (YRD), the Pearl River Delta (PRD), the Jing–Jin–Ji region (JJJ), and inland provincial capitals, and a few even above 50% (e.g., Shenzhen and Dongguan). Despite the net-outflow of laborers, many inland counties have more than 10% floating population residing in [3]. On the one hand, these floating population help mutual understanding between different regional cultures. On the other hand, however, the large composition of floating population denied of local rights may bring social inequality and instability [3]. Therefore, understanding the origin-destination pattern and the driving force of their migration is essential for central and local governments.

Earlier literature conceptualizes migration as a result of rational cost-benefit tradeoffs and mainly considers economic aspects [12,13]. However, recent works have begun to notice the weight of amenity-related factors in determining migration decisions [14,15,16,17]. Nevertheless, there has been no consensus on the importance of amenity aspects. Scholars have found that the extent of the effect of amenities on migration differs in the U.S. and in Europe [18], and the effect is heterogeneous across the life course [19]. We argue that this effect could also be changing over time. As mentioned earlier, of the 250 million floating population, about 50 million have college degrees. This better-educated floating population in the new Internet-based and highly electronic era would probably pay more attention to life quality, the spiritual world, and intellectual challenges, different to their older generation.

Our work contributes to the literature in the following ways. First, we study the migration behaviors of the skilled floating population, specifically the distribution and motivation of their migration. They are the neglected population in previous literature, but they are rising in the number and very special in the sense that they are well-educated but migrate to new cities where their local right is not afforded. Second, we use novel data source from a major online recruitment platform that offers unrivaled timeliness, great area coverage, large sample size, and richness of controls without loss of seriousness, as will be discussed in the data section. This data source enables us to complement previous research based on census data that is costly and updated only every ten years. Third, more than inter-provincial migration, we also study intra-provincial migrating too which is either neglected by previous research or not possible given the data. Fourth, we include both city-level amenity and wage variation, and person-level characteristics variation into our determinants of migration destination choices, making possible a comparison of relative importance of different attributes and the exhibition of heterogeneity among different types of migrants.

## 2. Literature

Few works have systematically studied the distribution of hukou origination cities. The few exceptions are [3,17]. Shi and Liu [3] use 2010 census data and through cartogram technique, they find a highly unbalanced distribution of hukou origination, with great heterogeneity both within and across key regions and provinces, and between coastal areas and inland borders. Zhang et al. [17] use data from a major recruitment platform and categorize hukou-origination cities into clusters according to their distinctive features driving human migration behavior. Most previous studies focus on hukou origination at province level [1,11,16,20,21]. However, in the context of China, cities could be quite different in various aspects even within a province. Therefore, city-level analysis is needed. Su et al. [22] perform a city-level analysis. They find that most migrants are from nearby cities, i.e., distance plays a dominant role. However, they fail to draw a bigger picture covering most cities (>200 cities) in China, as we do, due to small coverage (15 cities) of their source data. Moreover, they only focus on rural migrant workers, thus neglecting the growing population of skilled migrant workers. Unlike unskilled rural migrant workers, who are mostly from rural areas to cities, and western regions to east coast regions [7,23], we expect the hukou origination distribution of skilled migrant workers to be more diversified, in the sense that skilled migrant workers could have hukou originated from cities in east coast regions, driven by university attendance related relocation under the background of high education expansion, and attracted by more suitable job opportunities in the city where their graduating university is located. Therefore, we have our first proposition as follows:

**Proposition** **1.** *The hukou origination distribution of skilled migrant workers is more diversified than their unskilled counterparts.*

When choosing migration destinations, many aspects are taken into consideration. For instance, Su et al. [22] find a strong deterrent role played by distance between origination and destination. Both Liu and Shen [16] and Venhorst et al. [24] find that skilled migrants do not attach much value to the level of local unemployment rate, because skilled migrants are more competitive, risk-taking, and flexible than their less-educated counterparts in seeking new employment. Moreover, the reverse causality is raised by [25], who argue that the existence of a large number of risk-taking skilled migrants without a job in hand may swell the unemployment rate at the destination city. Some much earlier literature conceptualizes migration as a result of rational cost-benefit tradeoffs and mainly considers economic aspects [12,13].

However, recent trends of the floating behavior reveal some new factors. First, sizable lay-offs of workers from state-owned enterprises force local government to limit migrant access to jobs, especially for unskilled migrant. Second, technological development reduces the demand for unskilled workers [7]. Third, the rapid construction of high-speed railway lowers transportation costs greatly, not necessarily monetarily, but definitely in terms of time saving. To some extent, the upgrade of railway system is not monetarily friendly to unskilled migrant workers, but is quite friendly to their skilled counterparts because of time saving. Fourth, steady improvement of living standards in recent years has shifted the attention of migrants from merely economic aspects to amenity-related factors such as education, culture, and medical care [14,15,16,17]. As a result, unskilled migrant workers are slowly being replaced by machines in the traditional manufacturing industry, and gradually transferred to new service industries like express delivery. Despite working in different industries than their older generations, unskilled migrant workers are still in a situation with limited opportunities. On the other hand, skilled migrant workers, after taking certain examinations, could work as a public servant or public official, a high-end job according to most Chinese. They also benefit from the convenience of the rapid development of the high-speed railway system that connects cities in an unprecedented scale, speed, and punctuality. Therefore, new trends undoubtedly favor skilled migrant workers, and research on skilled migrant workers should gain more attention.

In view of this, we expect that in our study of skilled migrants, the distance and economic gap between origination and destination cities be important, but perhaps not so vital as in previous studies that focus on unskilled population. On top of this, we expect amenity to play a more important role for the skilled migrants. Literature shows that there has been no consensus on the importance of amenity aspects. Scholars have found that the extent of the effect of amenities on migration differs in the U.S. and in Europe [18], and the effect is heterogeneous across the life course [19]. In light of the status, we expect the relative importance of amenity to be heterogeneous, both across personal characteristics (age, education, etc.,) and across types of inter- or intra- provincial migrants. Therefore, we have our second proposition as follows:

**Proposition** **2.** *The distance and economic gap between origination and destination cities are important, but are not vital. Amenity-related factors will play an important role in determining migration behaviors, and the value attached to amenity will be heterogeneous across migrant types.*

Person-level socio-economic as well as demographic characteristics could also play an important role in determining the migration behavior. Previous literature mostly investigates at a macro-level, exploring migration matrix between regions [2,3,7,8,11]. Failure to consider the heterogeneity between migrating agents could result in not being able to detect some important and interesting regularities. Some exceptions include [1,2,5,22]. Specifically, they examine the effect of age on the attached value to amenity, consistent with the finding of heterogeneity across the life course by [19]. Therefore, in this study, we expect that age is non-negligible in determining migration: people above a certain age may care more about medical resources for their parents and educational resources for their offspring, etc. Apart from age, we also examine the effects of selectivity of graduating institutions, college majors, gender, and marital status. We expect unmarried, “hot majors”, and prestigious universities are associated with higher mobility. Therefore, we have our third proposition as follows:

**Proposition** **3.** *Person-level socio-economic as well as demographic characteristics could also play an important role in determining the migration behavior.*

## 3. Materials and Methods

We use resume data from zhaopin.com, one of the largest online job-matching websites in China. The reason we choose zhaopin.com is that it provides information on desired salary, self-view, and other variables we need. zhaopin.com was established in 1994, and its business covers the vast majority of cities in China. Its resume data contains variables such as age, gender, education experience, work experience, job intention (including desired salary), work status (working or out of work), residence city, workplace, hukou affiliation, etc. For this study, we choose a random sample that comprises job seekers of working age. Specifically, the sample consists of men and women between age 18 and 60, looking for a full-time job and that relevant variables are not missing. Our sample is a randomly selected one containing 80,000 resumes in June 2017.

Previous studies use data from RUMiC (rural-urban migration in China) or China’s 1% population sample survey [16,22]. The problem with the RUMiC data is that it focuses exclusively on poor, rural migrant laborers moving to the cities in search of jobs, eventually returning home to raise a family, thus neglecting the skilled migrants whose number is in rapid rise. Moreover, the RUMiC data only covers 15 cities, which is quite limited and could result in potential bias. The problem with 1% population sample survey or even census data is that they are updated only every 10 years and that they are largely highly official and thus very restrictive in terms of access and usage by average researchers. Moreover, the 1% population sample survey only contains information on inter-provincial migration [16], which makes it impossible to study intra-provincial migration. In fact, earlier literature mostly only focuses on inter-provincial migration [1,11,16]. Therefore, we attempt to fill the gap in studying intra-provincial together with inter-provincial migration.

Furthermore, as with census data in any country, there could be the issue of undercounting, where China census data has an undercount rate of 1.81% [8]. Given the very nature of “floating” population, the undercount rate of this group could be higher. Although there is no doubt that the census data is the most authoritative and perhaps the most reliable data source available, a trial on using the recruitment platform data is beneficiary, due to its unique advantages as follows.

First, data based on online recruitment platforms offers unrivalled timeliness, as it is almost updated on the go, therefore up-to-date and ready to fetch immediately. Second, our approach to data collection can offer large sample sizes and great area coverage, at very low marginal costs, at least in principle. In fact, our sample data include job seekers from more than 200 cities, making cross-city heterogeneity analysis possible, which could not be done by survey data other than national census due to high costs. Third, online recruitment platforms are typically more advantageous in the richness of controls. For instance, few surveys would collect information on college major and quality (selectivity), graduating institution, industry, occupation, employment history, and (desired) salaries, all at once. Fourth, unlike LinkedIn, resume data from zhaopin.com can only be seen by potential employers and is not available to the public. Therefore, the platform of zhaopin.com serves merely as a job market instead of social network. This guarantees that job seekers produce their resumes with seriousness and objectiveness, and without concerns about privacy issues. Moreover, recruiting firms need to pay about 20 yuan to view each resume. Although job seekers do not pay to use the platform, unserious job seekers are routinely removed by the AI system of the platform, to ensure that the platform keeps attracting recruiters.

Finally, annual and seasonal reports with aggregated and descriptive evidence based on data from recruitment platforms are released periodically, and cited by official media frequently (for example, people.com.cn, chinanews.com, sina.com.cn, sohu.com), largely due to the data’s timeliness and richness. Therefore, we claim that our data is reliable, and that research based on the data is an innovative and meaningful attempt to provide insights on human capital mobility between cities, at an even greater depth than the 1% sample data which only provide information on inter-provincial migration. In this sense, our study not only provides a timely complement to previous works, but also push research deeper from inter-provincial level to inter-city level.

To show the representativeness of our dataset, we compare it to the CFPS (China Family Panel Studies) dataset which was launched in 2010 by the Institute of Social Science Survey (ISSS) of Peking University China, designed to be nationally representative and widely used in academic research. To make the year comparable, we select the 2016 wave of the CFPS data. Note that CFPS data not only include skilled laborers but also unskilled laborers. In fact, three quarters of the population in CFPS are with education levels of junior high school or below. Together with those with a high school degree, they comprise almost 90% of the CFPS sample. Therefore, it is meaningful to compare only statistics of the well-educated subgroup from CFPS with that of our data sample. Appendix Table A1 shows that the average ages of the well-educated subgroups from CFPS are indeed remarkably consistent with that from zhaopin.com. We also tried to compare the average incomes and occupation distributions of the two datasets. Unfortunately, the CFPS data has 77.11% of income non-response rate and more than 90% with occupation unspecified, inhibiting potential comparisons. Moreover, it is frequently documented that income is underreported, especially by top income people in CFPS [26,27,28]. In view of this, we only compare the average ages. Yet average ages prove the accuracy of our dataset. We are not the first to use this dataset. In fact, the dataset from zhaopin.com is previously used by [17] to model a two-stage migration game with interaction between agents. Of course, this dataset has its own limitations. For instance, it may not cover the whole skilled floating population, especially those not temporally actively seeking new jobs. However, this is the best data source available to study the skilled floating population in terms of unrivaled timeliness, great area coverage, large sample size, and richness of controls without loss of seriousness.

Data of city-associated variables are obtained from China statistical yearbook 2013. Definitions and summary statistics of the data are shown in Appendix A Table A2 and Table A3.

The original sample data consists of 80,000 observations, including floating population and non-floating population. Some scholars treat non-migrants as potential migrants who choose their origin place [16,29], however, we believe that non-migrants may be very different from actual migrants in terms of personal traits. They may be the kind of people who take things as they are and never have considered migrating Therefore, treating them as “migrants” who choose their origin place as destination is not reasonable. The reason these studies keep those non-migrants might be because the sample size could be too small size after dropping non-migrants. We do not have this size issue with our data. After clearing out the non-floating population (i.e., people with local hukou status) and removing those without a college or equivalent degree, the sample size is 20,818.

We then order the destination cities in frequency and pick the top 26 cities whose frequencies are above 200 as top destinations. We then create a new binary variable that is by default one, which indicates whether the person actually migrates from its hukou registration place to the city of residence. After that, using a loop, we duplicate each observation 26 times with one city in the list of top destinations at a time, and change the binary variable to zero. In the loop process, if the city from the list of top destinations coincides with the individual’s city of residence, then this one piece of duplication is deleted. As a result, for each person, there are 26 or 27 observations, depending on whether or not his/her city of residence appears in the list of top destinations. The difference between the 26 or 27 observations is the values of the binary variable of migration, and the value of the city of potential destination and its associated city characteristics such as population, average wage and amenities.

We then use the conditional logit model to estimate individuals’ destination choice, assuming the error terms are independently and identically distributed with identical extreme value distribution. Specifically, the utility of individual *i* choosing city *j* is denoted by

(1) $$U_{ij}={x_{ij}}^{\prime }\alpha +{z_{i}}^{\prime }\beta +e_{ij}$$

where $x_{ij}$ represents the vector of destination-origin-related attribute variables, $z_{i}$ denotes the vector of individual personal characteristics, and $e_{ij}$ represents the error terms. Individual will choose a city as a destination if the utility from that choosing this city is not smaller than any other choice. Based on the extreme value distribution of the error terms, the conditional logit model gives the probability of individual *i* choosing destination *j* as

(2) $$\;\;\;\;\;\;P_{ij}=\exp({x_{ij}}^{\prime }\alpha +{z_{i}}^{\prime }\beta )/\overset{n}{\underset{j=1}{\sum }}\exp({x_{ij}}^{\prime }\alpha +{z_{i}}^{\prime }\beta )$$

where *n* = 26 or 27 depending on whether the individual’s destination city is within the list of top 26 destination cities.

## 4. Results

Table 1 exhibits the distribution of sources of hukou affiliations for laborers residing in representative cities in different regions of China. Specifically, we choose Beijing, Shanghai, Guangzhou, Shenzhen (the first-tier cities), as well as Hangzhou, Chengdu, Xi’an, and Shenyang as the representative emerging destination cities in Eastern Coastal, Western, Central, and Northeastern regions, respectively.

Table 1 shows that in all the representative cities except Shenyang and Xi’an, more than half of the skilled jobseekers do not have local hukou. Among them, Shenzhen (74.58%) ranks first with the highest percentage of skilled floating population, followed by Guangzhou (64.34%), Beijing (61.96%), Chengdu (57.51%), Hangzhou (56.4%), Shanghai (56.28%), Xi’an (49.58%), Shenyang (37.08%). It is remarkable that Hangzhou and Chengdu, as new emerging cities, have been comparable to the four 1st-tier cities (namely, Beijing, Shanghai, Guangzhou, Shenzhen) and even exceeds Shanghai in terms of the percentage of skilled floating population. This is consistent with anecdotal evidence more and more skilled population nowadays choose some emerging second-tier cities (or new first-tier cities) as their destination cities, apart from the traditional first-tier cities, presumably because of the higher living costs and lower probability of achieving local hukou in the traditional first-tier cities, and because of the flourishing development of the emerging industries in the new first-tier cities. This observation is consistent with [3].

At the city level, the distribution of hukou origination of the floating population is quite scattered, although most have registration from nearby cities with geographical proximity. The largest proportion of outside hukou registration place is 3.16% of Baoding for Beijing, 1.8% of Nantong for Shanghai, 5.13% of Maoming for Guangzhou, and similar for other representative cities.

At the province level as shown in Table 2, the commonality shared by the representative cities is that most skilled floating migrants are from neighboring provinces, forming regional clusters of human capital movements. The difference is that compared to other cities, the traditional first-tier cities have more proportions of skilled floating migrants from outside the province, making them more diversified migrant cities. In particular, Beijing, Shanghai, and Shenzhen have less than or around 50% of skilled floating migrants from within the province (here we treat Beijing and Shanghai as provinces since they are province-level municipalities under the direct control of the control government). Following Beijing, Shanghai, and Shenzhen, another two cities, namely Guangzhou and Hangzhou, have about three quarters of skilled job seekers from within the province. Finally, Chengdu, Xi’an, and Shenyang, representing the Western, Central, and Northeastern region respectively, have more than 90% of skilled job seekers from within the province. In sum, the traditional first-tier cities may serve as national attractions whereas emerging second-tier cities act as regional destinations.

**Figure 1:**
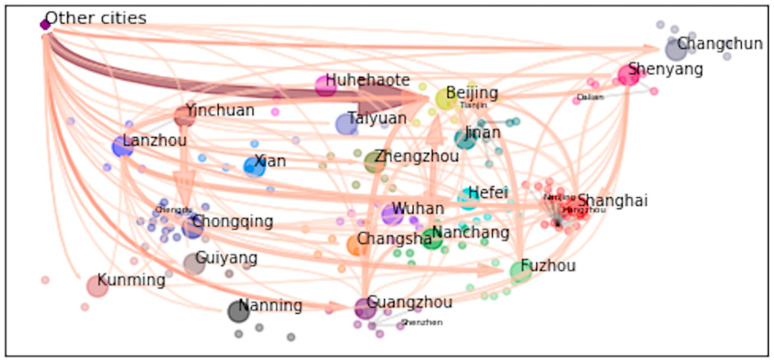
Migration flow network. The data is officially acquired from a database initiated by Minsheng Weekly, a subsidiary of the People’s Daily. The database by Minsheng has a random subsample of the resume database of zhaopin.com. The data is accessible for research purpose, but one has to apply for permission via their official website http://www.cnbo.tv or http://www.msweekydata.com, or email address cnbotv@163.com. The Chinese government ranks domestic universities and classifies them as “Project 985 Universities” and “Project 211 Universities”. As of 2018, there are 39 universities listed in “985 Project Universities”, as the first tier, and 112 universities listed in “211 Project Universities”, as the second-tier.

The above observations from Table 1 and Table 2 lend necessity to our research in two ways. First, “floating” skilled laborers compose a non-negligible proportion of skilled labor markets. Therefore, understanding what attracts them and keeps them floating on such a large scale is of increasing importance in today’s knowledge-migrating world. Second, both inter- and intra-provincial skilled migrants are not negligible. Table 1 and Table 2 show that large proportion of skilled migrants are from within the province, especially for non-first-tier cities; meanwhile, Figure 1 exhibits a large-scale and complex network of migration between provinces using the same data (the size of the arrow represents the value of the aggregated mean migration probability, see [17]). In this regard, both inter- and intra-provincial skilled migrants are not negligible. In fact, inter-provincial and intra-provincial skilled migrants may seek different goals and set different priorities in terms of amenity or employment. Therefore, further study is needed on differentiating the two types of skilled migrants. Failure to do so could lead to a biased understanding on the driving forces of the “floating” behaviors of skilled migrants, and thus result in less-than-optimal policies in directing the flow of human capital.

Table 3 presents the logit regression results with and without interaction with personal characteristics. The left panel shows the results for the model without interaction with personal characteristics, and the right panel shows the results for the model with interaction terms, where additional coefficients and standard errors of the interaction terms of Model 2 are separately presented in Table 4. The goodness of fit is measured by MaFadden’s $\rho^{2}$ [30] defined as $\rho^{2}=1-\ln\left(L\right)/\ln\left(L_{0}\right)$ where ln(L) is the maximum log likelihood for the model estimated and $\ln\left(L_{0}\right)$ is the maximum log likelihood for the same model with only a constant term. Both models have good ρ^2, indicating that important determinants of migration choice have been incorporated. The difference in the two ρ^2′s is small (0.5925 compared to 0.5833), probably because individual heterogeneity in making a migration destination choice is not significant. In other words, skilled floating migrants may share much commonality in value when making decisions regarding destination choice.

Most variables have expected signs. Specifically, the variable within has a positive sign, indicating that cities within the province are more attractive. This could be because of institutional and administrative barriers, for example, personal medical card issued by the government is freely usable within the province, whereas the card becomes unreadable outside the province. This provincial “border” effect could also be due to cultural differences such as the dialect and diet.

As expected, the variable dist has a negative sign, and its square term has a positive sign. This is consistent with conventional wisdom that the geographical distance (which is also related with cultural and institutional distances) plays a deterrent effect on the probability of migration, and that this effect is nonlinear, consistent with [22]. However, the coefficient for population of the destination city and population difference between origin and destination cities have unanticipated negative signs, which is not as predicted by the gravity theory. One possible explanation is that too dense population in the destination city may have crowding-out effects. Megacities like Beijing and Shanghai with a population size of more than twenty million are featured by high living costs and unsatisfactory traffic conditions, thus may inhibit the inflow of potential skilled migrants.

For employment variations, skilled migrants seem to value higher average wage, and do not care about higher unemployment rate. One explanation is that wage is the priority concern in the economic aspects for skilled migrants, whereas local unemployment rate is less relevant to them, partly because skilled migrants are more competitive, risk-taking, and flexible than their less-educated counterparts in seeking new employment [16,24]. Moreover, it is possible that the existence of a large number of risk-taking skilled migrants without a job in hand may swell the unemployment rate at the destination city [25].

Amenity-related variations also have significant impact on destination choice of the skilled migrants. The number of local libraries, expenditure on R&D in science and technology, the number of registered teachers in primary and secondary schools, and the number of hospital beds all have expected signs, and positively influence migration choices, indicating that skilled migrants attach importance to cultural atmosphere, investment in science, educational and medical resources. The variables of established building area and road length have negative signs, however. Our explanation is that maybe skilled migrants prefer more compact urban structure so as to avoid congestion and save transportation and time costs. In all, rather than the magnificence and appearance of physical construction, skilled migrants put more emphasis on the “soft power” of the destination cities.

The richness of the data enables us to further analyze the role of age, gender, college major, marital status and college selectivity in destination choice for the skilled migrants. Note that individual characteristics must be interacted with attributes of potential destinations in the model, because individual characteristics do not differ across potential destinations and will be crossed out in the calculation of probabilities in the logit model setting under the extreme value distribution as shown in Equation (2). Table 3 and Table 4 exhibit the results. Among them, the coefficients of the interaction terms with age and elite graduates are mostly significant, meaning age and elite graduates are important determinants.

Specifically, increase in age would lead to increase in the attached value to abundance in local educational as well as medical resources, higher average wage and lower unemployment rate, presumably because increase in age means increased probability of having had children, having taken on the responsibility of elderly caring, and thus bearing heavier economic burden (considering that the average age of our sample population is only 30.2, as in Table A2). These responsibilities carried by the migrants are also found by [1,2,3], etc. The coefficient of age * within has a negative sign, indicating that older job seekers may even prefer moving outside the province. This is probably because more senior job seekers are more flexible because they do better in finding their feet in a new city after a relatively longer time of effort and struggle in career, thus see a higher probability of achieving local hukou status and become permanent residents in the near future.

As to the college selectivity, job seekers graduated from elite institutions (defined as National Project 985 or 211 universities) are more likely to move away from their hometown, putting less emphasis on whether within home province or how far from hometown. This demonstrates that the elite class are more mobile and flexible than other classes, which may lead to “brain drain” of their hometown. Regarding college majors, we observe that people holding college degrees in STEM (science, technology, engineering, mathematics) and LEM (law, economics, finance, management) are more likely to relocate within their hukou province, presumably because it’s easier to find a satisfactory job with these majors.

Table 5 exhibits the relative importance of each set of variables, measured by the change in ρ^2^ if the set of variables is removed from the full model specification. The first columns present the results using the full sample including inter- and intra- provincial migration. The importance of amenity-related variables far exceeds the importance of employment-related variables, demonstrated by a much larger decrease in ρ^2^ (0. 0672) when removing amenity-related variables than the decrease in ρ^2^ (0. 0045) when removing employment-related variables. Moreover, the gravity-related variables and the interactions terms with personal characteristics are much less important determinants as measured by decrease in ρ^2^. Similarly, for the inter-provincial-only subsample, the relative importance of amenity outweighs that of employment. In contrast, the intra-provincial-only subsample exhibits prioritization of employment aspects over amenity considerations, yet with a relatively smaller gap in the two ρ^2^’s. A caveat should be kept in mind since ρ^2^’s are relatively small for the intra-provincial sample. Nevertheless, the recruiting platform data is the best source of data on China’s intra-provincial skilled floating population. The above observation indicated that the prioritization between amenity-related and pecuniary aspects is heterogeneous among different types of migrants. Therefore, failure to consider this in population-redistributing and talent-attracting policies might lead to undesirable outcomes.

## 5. Discussion

First, from the distribution aspect, in all first-tier cities and most emerging second-tier cities, more than half of the skilled jobseekers are floating, i.e., not afforded local rights. Although the hukou registration sources are quite scattered at the city level, most hukou originations are with nearby cities. At the province level, most floating population in emerging second-tier cities and provincial capitals are from surrounding provinces or with geographical proximity, whereas the traditional first-tier cities attract skilled floating population nationwide. This forms a big picture of the floating pattern of the skilled migrants as a few “super-star” national attractions surrounded by a number of “satellite” regional destinations. The regional inequalities and national hierarchies can be clearly detected.

Second, in regard to the determinants of migration at the origin-destination (OD) level, cities within the province are more attractive due to provincial “border” effect in the form of institutional, administrative, and cultural barriers. In terms of the geographical distance between origin and destination cities, this distance plays a nonlinear deterrent effect on the probability of migration. As to the attribute variables of the destination cities, “soft power” (e.g., expenditure on R&D., educational and medical resources, number of libraries) outweighs physical construction (e.g., road length). Taken together, cities closer and with similar cultural environment to hometown, and with nationally competitive educational and medical resources, rapid technological progress, and high cultural tolerance, are more attractive to skilled migrants. These cities will presumably grow to be where the future talents gather. Policy makers could possibly put more emphasis on these aspects for future resource allocation and urban planning, for a more balanced development of urban agglomeration.

Third, for the determinants of migration at the individual personal characteristics level, age and college selectivity matter. Specifically, increase in age would lead to increase in the attached value to abundance in local educational as well as medical resources, higher average wage and lower unemployment rate, presumably because increase in age means increased probability of having had children, having taken on the responsibility of elderly caring, and thus bearing heavier economic burden. Increase in age also implies higher probability of moving outside the province. This is probably because more senior job seekers are more flexible because they do better in finding their feet in a new city after a relatively longer time of effort and struggle in career, thus see a higher probability of achieving local hukou status and become permanent residents in the near future. In terms of college selectivity, people graduating from National Project 985 and 211 Universities are more mobile and flexible than their counterparts, which may lead to “brain drain” of their hometown and worsen the regional imbalance in development.

Fourth, we measure the relative importance of amenities and employment opportunities in the migration destination choice decisions of the floating population. We find that the importance of amenity-related variables exceeds the importance of employment-related variables. Similarly, for the inter-provincial-only subsample, the relative importance of amenity outweighs that of employment. In contrast, the intra-provincial-only subsample exhibits prioritization of employment aspects over amenity considerations, which means the prioritization between amenity-related and pecuniary aspects is heterogeneous among different types of migrants. Therefore, failure to consider the heterogeneity might lead to undesirable outcomes for the government in designing population redistribution, especially in the context of rapid urbanization in a knowledge-based world where the skilled labor force plays a vital role. In other words, “to float or not to float” is a question for the skilled migrants, and the answer to this question depends largely on whether to migrate within the province or outside the province, as well as on the amenity attributes of the destination city which are becoming more important nowadays.

Finally, a subtle point found in this paper is that the skilled floating migrants may share much commonality in value when making decisions regarding destination choice, as mentioned in the Results section. This phenomenon is probably uniquely seen in China, where obedience and uniformity are encouraged, and personality may be discouraged since pediatric education in its culture. This may result in remarkable herd effect when choosing destination cities, and lead to particular cities being over chased and overloaded, such as Beijing and Shanghai, whereas other cities suffering from brain drain.

Based on our findings, two important policy implications can be drawn: (1) In designing population redistribution policy, government should grant sufficient attention to the heterogeneity behind migrant characteristics (age and education background), migration types (namely, migrate within the province or outside the province) and the amenity attributes of the destinations. (2) The dominant importance of amenity attributes in deciding the inter-provincial floating destination suggests a polarization trend that cities winning in amenities will probably win skilled labor forces, especially from other provinces. This polarization trend implies inter-regional inequality, which should be taken into consideration in the design of regional development policy.

## 6. Conclusions

We use data from job-recruiting platforms to study the distribution patterns and migration determinants of skilled floating population in China. By fully utilizing the timeliness, large sample size, great area coverage, and rich controls, this paper is able to offer some insights on the intra- and inter- provincial migration behaviors of the skilled floating population in China that might otherwise be neglected if solely using the every-ten-year census data.

We find that most floating population in emerging second-tier cities and provincial capitals are from surrounding provinces or with geographical proximity, whereas the traditional first-tier cities attract skilled floating population nationwide. We also find that skilled migrants attach more value to educational and medical resources than to road and building constructions, especially for older migrants, as opposed to their younger counterparts without child- and elderly-caring responsibilities. Moreover, migrants graduating from more prestigious universities enjoy more flexibility in destination choices.

Last but not least, we find that the heterogeneity of attributes prioritizations differs between intra- and inter- provincial migrants. Intra-provincial skilled migrants put more value on employment opportunities than on amenity attributes, while their inter-provincial counterparts prioritize amenity over employment aspects. This could lead to important policy implications regarding the reversal of regional inequality and boosting urban development. Our work emphasizes the importance of studying the skilled migrant worker population who is growing dramatically in size. Future work could use data over time and thus show a bigger picture by demonstrating the trend of migration behaviors by the skilled.

## Figures and Tables

**Table 1:** hukou origination **cities** of representative destination cities (in percentage).

Beijing		Shanghai		Guangzhou		Shenzhen		Xi’an		Chengdu		Hangzhou		Shenyang	
Beijing	38.04	Shanghai	43.72	Guangzhou	35.66	Shenzhen	25.42	Xi’an	50.42	Chengdu	42.49	Hangzhou	43.60	Shenyang	62.92
Baoding	3.16	Nantong	1.80	Maoming	5.13	Jieyang	3.32	Weinan	7.24	Nanchou	5.65	Shaoxing	3.28	Jinzhou	3.13
Tianjin	2.15	Yancheng	1.19	Zhanjiang	4.58	Meizhou	2.93	Xianyang	6.13	Dazhou	3.34	Wenzhou	2.67	Chaoyang	2.7
Shijiazhuang	1.85	Zhoukou	1.00	Shantou	4.14	Shanwei	2.74	Baoji	5.29	Neijiang	2.61	Quzhou	2.13	Fushun	2.38
Handan	1.82	Anqing	0.95	Meizhou	3.71	Heyuan	2.25	Shangluo	3.62	Mianyang	2.61	Jinhua	1.98	Tieling	2.28
Zhangjiakou	1.74	Hefei	0.83	Shaoguan	2.73	Zhanjiang	1.96	Yan’an	3.06	Guang’an	2.49	Shaorao	1.75	Huludao	1.72

**Table 2:** hukou origination **provinces** of representative destination cities (in percentage).

Beijing		Shanghai		Guangzhou		Shenzhen		Xi’an		Chengdu		Hangzhou		Shenyang	
Beijing	38.04	Shanghai	43.72	Guangdong	77.62	Guangdong	52.76	Shan’xi	93.47	Sichuan	90.51	Zhejiang	72.32	Liaoning	95.00
Hebei	12.07	Jiangsu	8.57	Hunan	3.83	Hunan	9.27	Gansu	1.37	Chongqing	2.61	Jiangxi	4.8	Heilongjiang	1.67
Heilongjiang	2.95	Anhui	4.45	Hubei	3.43	Hubei	8.56	Sichuan	1.03	Guizhou	0.72	Anhui	4.61	Jilin	1.16
Liaoning	2.71	Zhejiang	2.43	Jiangxi	2.62	Jiangxi	5.88	Shanxi	1.03	Guangdong	0.58	Jiangsu	3.23	Hebei	0.53
Tianjin	2.15	Hubei	2.29	Sichuan	2.02	Sichuan	4.81	Guangdong	0.69	Gansu	0.58	Hubei	2.58	Shandong	0.26
Shandong	1.80	Henan	2.02	Guangxi	1.41	Guangxi	2.5	Jilin	0.34	Shan’xi	0.58	Hunan	1.48	Jiangsu	0.26

**Table 3:** Regression results.

Variables	Model 1: No Interaction Terms		Model 2: With Interaction Terms	
	Coefficient	s.e.	Coefficient	s.e.
Within	2.164 ***	(0.035)	3.693 ***	(0.179)
Gravity-Related	-	-	-	-
Dist	−2.856 ***	(0.058)	−3.065 ***	(0.137)
Dist^2^	0.790 ***	(0.025)	0.789 ***	(0.025)
Population	−0.744 ***	(0.203)	−0.704 ***	(0.206)
Popu_diff	−0.040 *	(0.025)	−0.061 **	(0.025)
Job-Related	-	-	-	-
Page	0.514 ***	(0.036)	0.234 ***	(0.058)
Unemployed	0.142 ***	(0.035)	0.382 ***	(0.078)
Amenity-Related	-	-	-	-
Building_area	−0.069 ***	(0.017)	−0.066 ***	(0.017)
Library	0.007 *	(0.004)	0.133 ***	(0.020)
Road_len	−0.734 ***	(0.067)	−0.723 ***	(0.068)
Sci_tech_expenditure	0.033 ***	(0.001)	0.033 ***	(0.001)
Teachers_num	0.049 ***	(0.006)	0.006	(0.010)
Beds_num	0.559 ***	(0.018)	0.422 ***	(0.046)
N	514,483	-	511,106	-
$\rho^{2}$	0.5833	-	0.5925	-

Note: ***, **, * denote significance levels of 1%, 5% and 10%.

**Table 4:** regression results with interaction terms of Model 2.

Variables	Coefficient	s.e.		Coefficient	s.e.		Coefficient	s.e.
Age *			LEM_Major *			Married *		
Within	−0.054 ***	(0.006)	Within	0.172 **	(0.073)	Within	−0.012	(0.087)
Dist	0.006	(0.004)	Dist	0.016	(0.055)	Dist	−0.094	(0.066)
Wage	0.009 ***	(0.002)	Wage	−0.032 *	(0.019)	Wage	0.023	(0.024)
Unemployed	−0.006 ***	(0.002)	Unemployed	−0.026	(0.030)	Unemployed	−0.038	(0.035)
Library	−0.004 ***	(0.001)	Library	0.014 *	(0.008)	Library	−0.012	(0.009)
Teachers_num	0.002 ***	(0.000)	Teachers_num	−0.008 *	(0.005)	Teachers_num	−0.013 *	(0.007)
Beds_num	0.004 ***	(0.002)	Beds_num	−0.002	(0.017)	Beds_num	0.031	(0.021)
Male *			STEM_major			Grad_Elite *		
Within	−0.081	(0.064)	Within	0.186 ***	(0.065)	Within	−0.158 *	(0.081)
Dist	−0.012	(0.050)	Dist	0.012	(0.050)	Dist	0.174 ***	(0.058)
Wage	0.009	(0.017)	Wage	−0.015	(0.017)	Wage	−0.018	(0.021)
Unemployed	0.025	(0.027)	Unemployed	−0.008	(0.027)	Unemployed	−0.183 ***	(0.033)
Library	0.003	(0.007)	Library	0.009	(0.007)	Library	−0.003	(0.009)
Teachers_num	0.002	(0.004)	Teachers_num	0.001	(0.004)	Teachers_num	−0.008	(0.005)
Beds_num	−0.015	(0.015)	Beds_num	−0.020	(0.016)	Beds_num	0.065 ***	(0.019)

Note: ***, **, * denote significance levels of 1%, 5% and 10%.

**Table 5:** Change in $\rho^{2}$ for different model specifications and subsamples.

	Full Sample		Only Inter-Provincial Sample		Only Intra-Provincial Sample	
Model	$\rho^{2}$	$\Delta \;\mathrm{in}\;\rho^{2}$	$\rho^{2}$	$\Delta \;\mathrm{in}\;\rho^{2}$	$\rho^{2}$	$\Delta \;\mathrm{in}\;\rho^{2}$
Full Model	0.5925	-	0.6607	-	0.1683	-
Personal	0.5833	−0.0092	0.6554	−0.0053	0.1469	−0.0214
Gravity	0.5881	−0.0044	0.6501	−0.0106	0.1207	−0.0475
Employment	0.5880	−0.0045	0.6589	−0.0018	0.1498	−0.0185
Amenity	0.5253	−0.0672	0.5905	−0.0702	0.1603	−0.0080

## Appendix A

**Table A1:** Comparison of datasets from CFPS_2016 and zhaopin.com.

Education	CFPS_2016 Full Sample		Our Sample from zhaopin.com	
Education qualifications	Percent	Average age	Percent	Average age
Junior High School or below	75.47%	48.96	0.71%	31.13
High School	14.34%	39.63	9.63%	31.85
Some College	5.99%	33.96	40.55%	31.20
Bachelor	3.78%	33.84	48.07%	30.92
Master	0.39%	32.91	1.02%	33.17
Doctor	0.03%	31.44	0.03%	40.20
N	34,992	-	20,818	-

**Table A2:** Definition of city-level variables.

Category	Variable Name	Definition
Province Border	Within	Within Province
Gravity-Related	Dist	Distance between OD Cities
	Dist^2^	Distance Squared
	Population	Population at Destination City
	Popu_diff	Population Difference b/w OD Cities
Job-Related	Wage	Average Wage at Destination City
	Unemployed	Unemployment Rate at Destination City
Amenity-Related	Building Area	Built-up Area
	Library	Number of Libraries
	Road Len	Length of All Roads
	Sci_tech_expenditure	Expenditure on R&D in Science and Technology
	Teachers_num	Number of Registered Primary and Secondary Schools
	Beds_num	Number of Hospital Beds

**Table A3:** Definition and summary statistics of personal characteristics.

Variable	Definition	Mean	Std. Dev.	Min	Max
Age	Age	30.202	5.959	20	60
Eduy	Years of Education	15.604	0.570	15	21
Married	Married	0.237	0.425	0	1
LEM_Major	Major in Law, Economics, Management	0.277	0.448	0	1
STEM_Major	Major in Science, Technology, Engineering, Mathematics	0.449	0.497	0	1
Male	Male	0.517	0.500	0	1
N	Observation Number = 20,818	-	-	-	-
